# Viscoelastic testing reveals normalization of the coagulation profile 12 weeks after severe COVID-19

**DOI:** 10.1038/s41598-021-92683-1

**Published:** 2021-06-25

**Authors:** Abakar Magomedov, Daniel Zickler, Stoyan Karaivanov, Annika Kurreck, Frédéric H. Münch, Julian Kamhieh-Milz, Caroline Ferse, Andreas Kahl, Sophie K. Piper, Kai-Uwe Eckardt, Thomas Dörner, Jan Matthias Kruse

**Affiliations:** 1 Department of Nephrology and Medical Intensive Care, Charité-Universitätsmedizin Berlin, Augustenburger Platz 1, 13353 Berlin, Germany; 2 Department of Hematology and Oncology, Charité-Universitätsmedizin Berlin, Berlin, Germany; 3 Department of Transfusion Medince, Universitätsmedizin Berlin, Berlin, Germany; 4 Wimedko GmbH, Manfred-von-Richthofen Str. 15, 12101 Berlin, Germany; 5 Institute of Biometry and Clinical Epidemiology, Charité-Universitätmedizin Berlin, Berlin, Germany; 6 Berlin Institute of Health (BIH), Anna-Louisa-Karsch 2, 10178 Berlin, Germany; 7 Department of Rheumatology und Clinical Immunology, Charité-Universitätsmedizin Berlin, Berlin, Germany; 8 Deutsches Rheumaforschungszentrum (DRFZ) Berlin, Berlin, Germany

**Keywords:** Infectious diseases, Respiratory tract diseases

## Abstract

COVID 19 is associated with a hypercoagulable state and frequent thromboembolic complications. For how long this acquired abnormality lasts potentially requiring preventive measures, such as anticoagulation remains to be delineated. We used viscoelastic rotational thrombelastometry (ROTEM) in a single center cohort of 13 critical ill patients and performed follow up examinations three months after discharge from ICU. We found clear signs of a hypercoagulable state due to severe hypofibrinolysis and a high rate of thromboembolic complications during the phase of acute illness. Three month follow up revealed normalization of the initial coagulation abnormality and no evidence of venous thrombosis in all thirteen patients. In our cohort the coagulation profile was completely normalized three months after COVID-19. Based on these findings, discontinuation of anticoagulation can be discussed in patients with complete venous reperfusion.

## Introduction

SARS-CoV-2 is a single stranded RNA virus belonging to the coronavirus family. It causes coronavirus disease 2019 (COVID-19) which often can take an asymptomatic course but can also result in substantial severe manifestations, such as acute respiratory failure, acute kidney failure, multi-organ dysfunction and death. It has led to a global pandemic with over 2.9 million attributable death toll to date^1,2^.

While severe respiratory failure seems to be the most frequent cause of death, other complications like acute kidney injury, cardiac and neurologic involvement seem to occur more frequent than initially expected^3–6^.

From the beginning of the pandemic, a high incidence of thromboembolic complications was reported in patients with COVID-19 and autopsy findings confirmed a high rate of local thrombosis and embolic events in the pulmonary and systemic circulation associated with the disease^7–11^. Laboratory markers revealed hyperinflammation linked to a hypercoagulable status with markedly elevated levels of fibrinogen, d-dimers and thrombocytosis. Numerous haemostatic tests performed in ICU patients with COVID-19 have shown, namely viscoelastic tests (ROTEM, TEG), Thrombin Generation (reference 1, 2, 3) and Clot Waveform analysis (reference 4, 5) have demonstrated a hypercoagulable state^12–16^.

In our intensive care units we performed viscoelastic testing and noted severely impaired fibrinolysis as a characteristic of procoagulation in COVID-19^17^. In this context the term fibrinolytic shutdown has been proposed^18^.

These findings led to intensive discussions about the pros and cons of systemic anticoagulation, use of available drugs and intensity of systemic anticoagulation^19–23^. The question for how long survivors of the disease should receive subsequent anticoagulation as secondary prophylaxis is unclear so far. Long term data regarding the question whether the hypercoagulable state persists after clinical cure of the disease are currently lacking^23^.

Here we present observational data of 13 critically ill patients with COVID-19 who presented at the post Intensive Care clinic of a tertiary care university hospital 3 months after discharge from ICU.

## Methods

### Follow-up cohort

Out of 41 Covid-19 patients admitted to our ICUs between March 25th and May 11th 2020, 29 patients survived^17^. All of these were invited for a follow-up visit. Thirteen patients came back to the follow-up visit after 3 months.

### Anticoagulation

Upon admission on the ICU, all COVID patients were treated with therapeutic anticoagulation using intermediate doses of unfractionated heparin or argatroban with a aPTT goal of 50–55 s. Nevertheless, nine out of thirteen patients developed thromboembolic complications during their stay on the intensive care unit (ICU). These patients were then anticoagulated using higher doses of unfractionated heparin or argatroban with a targeted partial thromboplastin time of 60–80 s, respectively. Upon transfer to rehabilition units the continuation of this therapy regimen was recommended followed Rivaroxaban 20 mg per day until the first visit to the follow up clinic 12 weeks after discharge from the ICU.

### Coagulation tests

After admission to our ICUs, routine viscoelastic tests were performed with citrated blood by using a ROTEM sigma point-of-care device (Tem International, Munich, Germany)^24^. In each patient, intrinsically (contact activation, INTEM) and extrinsically (tissue factor activation, EXTEM) activated test assays were performed to analyze the clot dynamics in both coagulation pathways. Furthermore, FIBTEM and HEPTEM were performed. For FIBTEM thrombocytes are inactivated with Cytochalasin D to allow isolated evaluation of fibrinogen in clot firmness. For HEPTEM heparinase is added. The heparin effect was determined by comparing the clotting time of the INTEM with the clotting time of the HEPTEM.

**Figure 1:**
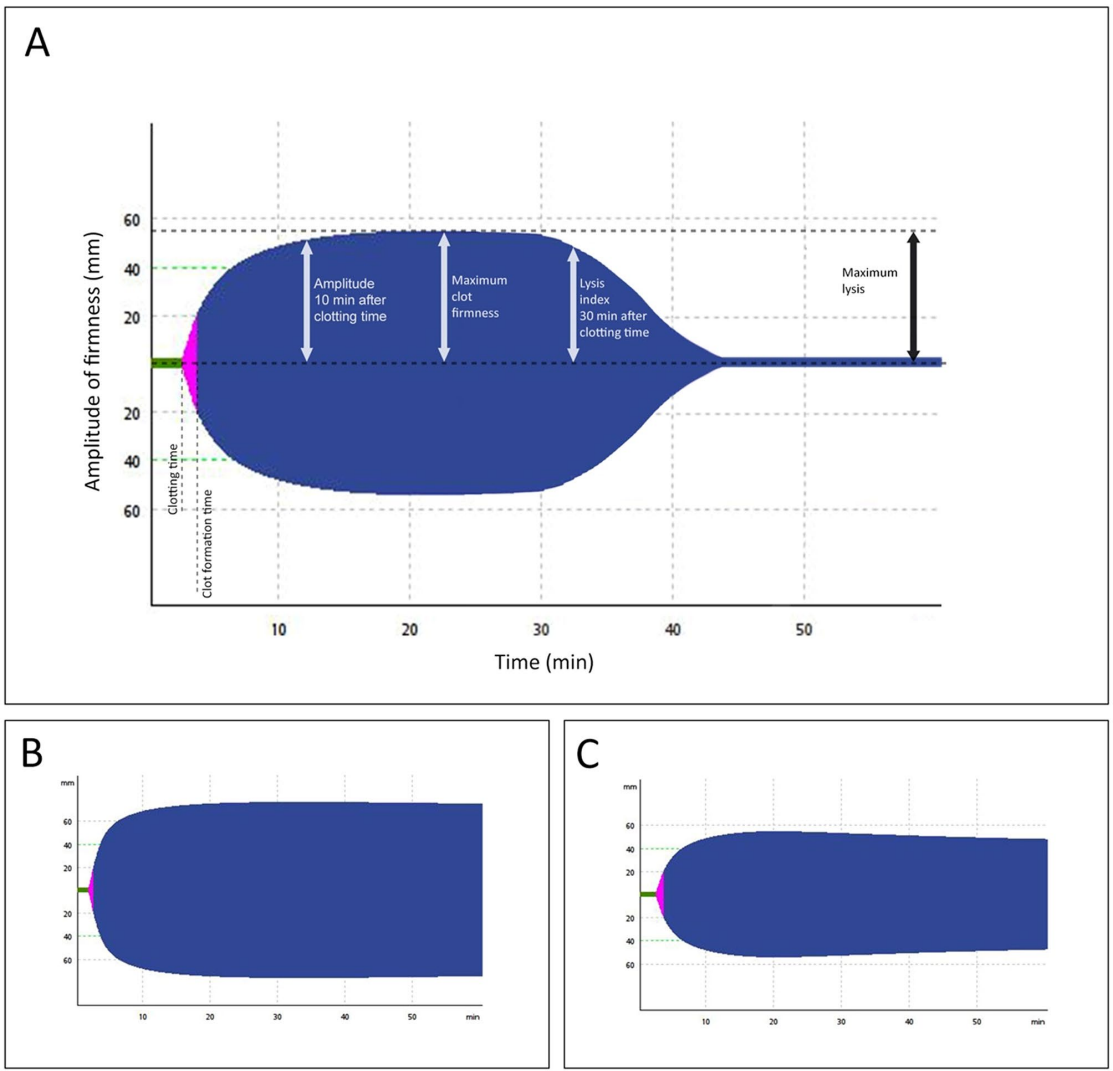
(**A**) Shows all measured values in ROTEM analysis, including clotting time (CT [s]), clot formation time (CFT [s]), maximum clot firmness (MCF [mm]) and maximum lysis (ML [%(range)]). (**B**) Describes a fibrinolysis shutdown pattern (increased MCF, low ML) in a COVID-19 patient with a thromboembolic event; the clot amplitude remains unchanged until the end. (**C**) Shows a clot profile with reduced MCF and increased ML in a patient during the follow-up presentation.

The following ROTEM variables were analyzed: clotting time defined as the time until initiation of clotting; clot formation time (seconds until a clot strength reaches 20 mm), reflecting the kinetics of clot formation; maximum clot firmness (MCF) defined as the maximum amplitude of clot firmness; maximum lysis (ML) in percent (%) defined as the difference between MCF and the lowest clot amplitude after MCF, reflecting fibrinolytic activity (Fig. 1).

Additional routine laboratory tests carried out according to standardized protocols comprised haemoglobin concentration, white blood cell count, platelet count, prothrombin time (PT), International Normalized Ratio (INR), activated partial thromboplastin time (aPTT), d-dimers, fibrinogen, Interleukin 6, CRP and Ferritin (see Table 2).

### Ultrasound assessments

During the ICU stay we performed ultrasound examinations in all patients (GE Vivid S70 ultrasound machine with a 9L-D probe) to screen for deep venous thrombosis, focusing on the jugular, subclavian, brachial, femoral and popliteal veins upon admission to our ICU and subsequently at least once a week.

The same examinations were performed as part of the 3 months follow-up visit.

### Post-ICU-follow-up evaluations

All patients were invited for follow-up visits scheduled 3 months after discharge from the ICU. Follow-up visits took place at our post-ICU outpatient department and followed a standardized procedure including a medical consultation, ultrasound examination of the large vessels as described above and routine laboratory work up, including vioscoelastic tests.

### Ethics

The study was approved by the ethics committees of Charité—Universitätsmedizin Berlin (EA4/115/20) and was in compliance with the Declaration of Helsinki. Consent to participate was not applicable due to the retrospective nature of the study. Informed consent was exempted by the ethics committee of the Charite Universitätsmedizin Berlin (EA4/115/20).

### Statistics

Statistical evaluations were performed with IBM SPSS Statistics Version 26 (New York, USA) and GraphPad Prism (version 8.4.3; GraphPad Software, San Diego, CA, USA). Descriptive variables were given as median with limits of the interquartile range (IQR) for continuous variables or as absolute and relative frequencies for categorical variables.

Continuous data were mostly right skewed. Therefore, Wilcoxon signed rank test was used to compare changes in continuous variables between ICU stay and 3 months follow-up evaluation. A two-sided significance level of 0.05 was applied without adjustment for multiple comparison. All p-values constitute exploratory data analyses and do not allow for confirmatory generalization of results.

## Results

Out of 41 Covid-19 patients admitted to our ICUs between March 25th and May 11th 2020, thirteen patients came back to the follow-up visit after three months. Out of 13 patients, 9 were male with a median age of 60 [IQR 53–67] years and a median BMI of 29.6 [IQR 27.8–33.9].

Their median SOFA score was 10.0 [IQR 5.3–11.8] points and their median APACHE II was 32.5 [IQR 25.3–34.0] points. Eleven patients required mechanical ventilation (85%), whereas extracorporeal membrane oxygenation was required in five (39%). Nine patients developed acute renal failure requiring continuous renal replacement therapy (69%). Median length of stay in the intensive care unit was 45 [IQR 30–65] days. (Table 1).

**Table 1:** Baseline characteristics of patients with COVID-19 infection.

	Whole cohort (n = 41)		No follow-up-cohort (n = 28)		Follow-up cohort (n = 13)		P follow-up vs. no follow-up
Age (years, (median, [IQR]))	67	[56.5–76.5]	69.5	[59–78]	60	[53–66.5]	0.019
Gender, male (n, %)	35	85.4%	26	92.9%	9	69.2%	ns
BMI, kg/m^2^ (median, [IQR])	28.0	[25–32.7]	27.8	[24.3–31.1]	29.6	[27.8–33.5]	ns
Duration of ICU stay, days (median, [IQR])	39.0	[24–52.5]	28.5	[24–47.5]	46	[30–62]	ns
Death during ICU stay (n, %)	11	26.8%	11	39.3%			
Thromboembolic events (n, %)	24	58.5%	15	53.6%	9	69.2%	ns
Intubation (n, %)	36	87.8%	25	89.3%	11	84.6%	ns
ECMO (n, %)	10	24.4%	5	17.9%	5	38.5%	ns
CRRT (n, %)	22	53.7%	13	46.4%	9	69.2%	ns
SOFA-Score (median, [IQR])	9	[6.5–11.5]	8.5	[6.25–11]	10	[6.25–12.0]	ns
SIC-Score (median, [IQR])	3	[2–4]	3	[2–4]	3	[2–4]	ns
APACHE-Score (median, [IQR])	28	[22–33]	26	[22–32]	31.0	[25.5–34]	ns
Follow up days					100	[63.5–108.50]	
**Preexisting conditions**							
Coronary artery disease (n, %)	9	22%	7	25%	2	15.4%	ns
Hypertension (n, %)	28	68.3%	18	64.3%	10	76.9%	ns
Diabetes mellitus/insulin resistance (n, %)	13	31.7%	8	28.6	5	38.5%	ns
Chronic kidney disease (n, %)	7	17.1%	5	17.9%	2	15.4%	ns
Chronic dialysis (n, %)	1	2.4%	1	3.6%	0	0%	ns
Lung disease (n, %)	10	24.4%	7	25%	3	23.1%	ns

*ECMO* Extracorporeal membrane oxygenation, *SOFA* sequential organ failure assessment, *CRRT* continuous renal replacement therapy, *SIC* Sepsis-Induced Coagulopathy Score, *APACHE* acute physiology and chronic health evaluation.

### Laboratory parameters

The laboratory values are displayed in Table 2 and showed distinctive changes between the ICU and post-discharge timepoints regarding inflammatory and coagulation parameters.

**Table 2:** The laboratory and ROTEM values at ICU presentation and 3 months follow-up.

	ICU (N = 13)		Follow-up (N13)		p value
	Median	[IQR]	Median	[IQR]	
**Laboratory variables (normal values)**					
Haemoglobin (12.5–17.2 g/dL)	9.9	[8.7–11.0]	13.6	[10.6–14.1]	0.006
White blood cells (3.5–10.5/nl)	12.8	[8.0–13.6]	7.1	[5.3–9.1]	0.023
Platelet count (150–370/nl)	142.0	[116.0–271.5]	224.0	[193.5–237.0]	0.263
Prothrombin time (70–130%)	79.0	[64.0–86.5]	85.0	[78.5–96.5]	0.196
INR (0.9–1.25)	1.2	[1.1–1.4]	1.1	[1.0–1.2]	0.173
aPTT (26–40 s)	56.4	[49.2–62.0]	36.8	[34.4–43.7]	0.033
d-dimers (< 0.5 mg/l)	4.8	[3.9–7.8]	0.4	[0.3–0.8]	0.001
Fibrinogen (1.6–4 g/l)	6.7	[4.7–8.3]	3.6	[2.9–4.5]	0.006
IL-6 (< 7 ng/l)	173.0	[68.0–358.0]	2.9	[2.1–6.3]	0.002
CRP (< 0.5 mg/l)	191.0	[121.0–314.4]	3.0	[1.6–5.4]	0.001
Ferritin (30–400 µg/l)	2408.7	[1441.5–5280.5]	190·4	[108.6–288.2]	0.002
**ROTEM variables (reference ranges)**					
FIBTEM CT (43–69 s)	92.0	[86.5–104.5]	99.0	[68.0–110.5]	0.463
FIBTEM CFT (n/a s)	101.0	[54.5–200.5]	198.0	[57.5–559.0]	0.225
FIBTEM A10 (9–24 mm)	29.0	[24.0–35.5]	15.0	[13.0–21.5]	0.002
FIBTEM MCF (9–25 mm)	32.0	[27.0–39.0]	16.0	[14.0–24.0]	0.002
EXTEM CT (42–74 s)	88.0	[83.5–101.0]	79.0	[62.5–100.5]	0.039
EXTEM CFT (46–148 s)	54.0	[42.0–64.5]	64.0	[48.5–82.5]	0.208
EXTEM A10 (43–65 mm)	66.0	[63.0–68.5]	59.0	[54.5–62.5]	0.033
EXTEM MCF (49–71 mm)	73.0	[71.5–76.0]	66.0	[63.5–70.0]	0.004
INTEM CT (137–246 s)	215.0	[187.5–258.0]	189.0	[176.5–201.5]	0.019
INTEM CFT (40–100 s)	56.0	[50.5–60.5]	71.0	[52.5–79.5]	0.075
INTEM A10 (44–68 mm)	64.0	[60.0–70.5]	57.0	[53.5–59.5]	0.006
INTEM MCF (52–72 mm)	73.0	[70.0–76.5]	63.0	[59.5–67.5]	0.003
ML EXTEM (0–18%)	3.0	[3.0–5.0]	8.0	[6.0–12.0]	0.002
ML INTEM (0–12%)	2.0	[2.0–4.0]	8.0	[6.0–13.0]	0.002

Unless values are designated as maximum values during the ICU stay, these parameters were determined on the day, when ROTEM analysis was performed, after admission to our ICUs.

*CT* Clotting time, *CFT* clot formation time, *MCF* maximum clot firmness, *ML* maximum lysis^55^.

**Figure 2:**
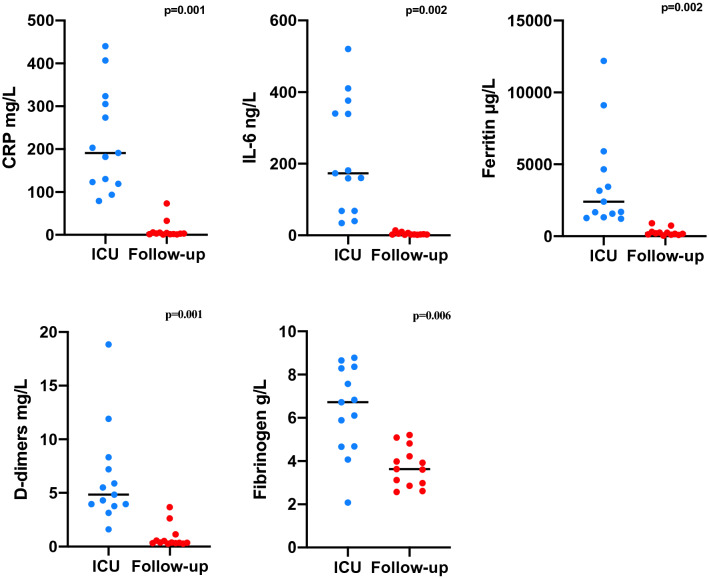
Comparison of inflammatory markers during ICU stay and follow-up visit (n = 13, Wilcoxon signed rank test).

In terms of the measurements of coagulation values, patients during their initial ICU admission had significantly elevated levels of d-dimers (4.8 mg/l [IQR 3.9–7.8] vs. 0.4 mg/l [IQR 0.3–0.8], p = 0.001) and fibrinogen (6.7 mg/l [IQR 4.7–8.3] vs. 3.6 mg/l [IQR 2.9–4.5], p = 0.006) compared to their follow-up measurements (Fig. 2). Moreover, the median of fibrinogen and d-dimers levels returned to a normal level at the follow-up visit (Table 2). aPTT was significantly prolonged during the ICU stay (56.4 s [IQR 47.6–62.0] *vs.* 36.8 s [IQR 34.4–43.7], p = 0.033), whereas prothrombin time and INR revealed no significant difference between measurement time points. CRP (191.0 mg/l [IQR 121.0–314.4] vs. 2.9 mg/l [IQR 2.1–6.3], p = 0.001), Ferritin (2408.7 µg/l [IQR 1441.5–5280.5] vs. 190.4 µg/l [IQR 108.6–288.2], p = 0.002) and IL-6 (177 ng/l [IQR 68.0–358.0] vs. 2.9 ng/l [IQR 2.1–6.3], p = 0.02) were significantly elevated during the ICU stay.

**Figure 3:**
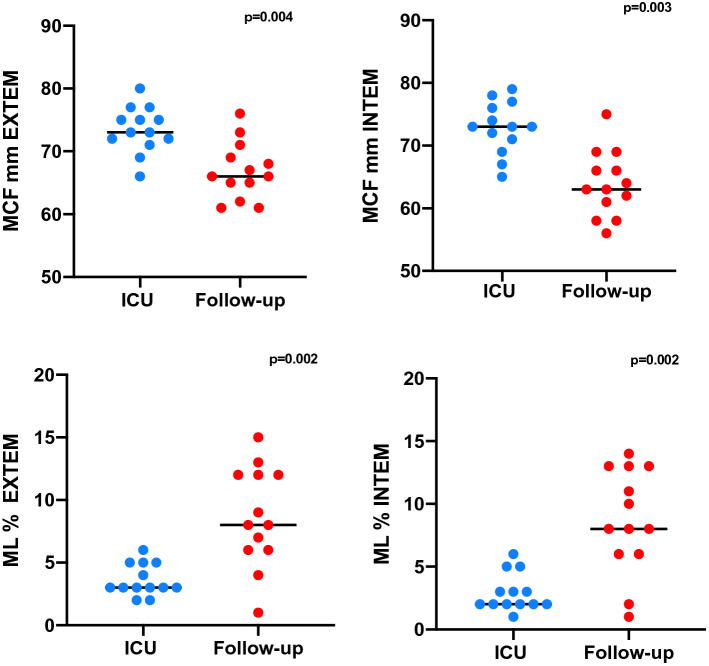
Comparison of coagulation parameters during ICU stay and follow-up visit (n = 13, Wilcoxon signed rank test).

Notably, ROTEM analyses showed substantial changes between the measurements between the ICU stay and follow-up visit. (Fig. 3) Maximum clot firmness decreased significantly with median values from 73 mm [IQR 70.0–76.5] to 63.0 mm [IQR 59.5–67.5] (p = 0.003), in INTEM; from 73 mm [IQR 71.5–76] to 66.0 mm [IQR 63.5–70.0] (p = 0.004) in EXTEM and from 32 mm [IQR 27.0–39] to 16 mm [IQR 14–24] (p = 0.002), in FIBTEM.

Clot formation time in EXTEM und INTEM were longer at the follow up visit compared to ICU measurements, but these changes were not statistically significant. In contrast to the ICU values, the maximum Lysis (ML) in INTEM and EXTEM increased significantly until 3 months follow-up (ML INTEM from 2% [IQR 2–4] to 8% [IQR 6–13] at Follow-Up; p = 0.002; ML EXTEM median from 3% [IQR 3–5] to 8% [IQR 6–12] at Follow-Up; p = 0.002) marking a substantial normalization of the fibrinolytic capacity which was markedly impaired at the initial assessments.

### Thromboembolic events

During ICU stay, nine patients developed thromboembolic complications. In this regard, two patients developed pulmonary embolisms, while lower-extremity deep venous thrombosis was found in seven patients.

At 3 month follow-up, there was no sonographic evidence of thrombosis in any of the patients indicating complete recanalization of the prior occluded veins (see Table 3).

**Table 3:** Results of the ultraononographic examinations at 3 month follow up.

Age	Gender	Thromboembolic event	Follow-up sonogram
51	F	Left femoral vein	No evidence of thrombosis
59	M	Left femoral vein	No evidence of thrombosis
60	M	Left popliteal vein	No evidence of thrombosis
67	M	Left and right popliteal vein	No evidence of thrombosis
64	F	Bilateral central pulmonary embolism	No evidence of thrombosis
56	M	Right femoral and external iliac vein	No evidence of thrombosis
47	M	Bilateral central pulmonary embolism	No evidence of thrombosis
38	F	Femoral vein and ecmo cannula clotting	No evidence of thrombosis
75	M	Right internal jugular vein	No evidence of thrombosis
68	M	No thromboembolic event	No evidence of thrombosis
62	M	No thromboembolic event	No evidence of thrombosis
55	F	No thromboembolic event	No evidence of thrombosis
66	M	No thromboembolic event	No evidence of thrombosis

## Discussion

We report the data of thirteen critically ill patients with COVID-19, who initially required ICU admission with a severely hyperinflammatory and hypercoagulable state characterized by high levels of d-dimers and fibrinogen and a markedly increased clot firmness consistent with impaired fibrionolysis. While a hypercoagulable state was noted during their stay on the ICU, on their first visit in the ICU-follow-up clinic three months after discharge, they presented with normalized markers of inflammation and coagulation. Fibrinolytic activity and clot firmness had returned to normal values consistent with the reversible nature of the initial hypofibrinolysis. All patients analyzed showed normal d-dimer levels reflecting normalized turnover of the coagulation system also taken as surrogate of recurrence risks. Following normalization of laboratory values and viscoelastic parameters anticoagulation has been discontinued in all patients.

Venous and arterial thrombembolism contribute significantly to morbidity and mortality in COVID-19^7–11,25,26^. The nature of COVID-19 coagulopathy appears to be complex and the exact mechanisms still have to be elucidated. In contrast to septic coagulopathy thrombocytopenia seems to be a rare finding and only few patients with COVID-19 meet the criteria for disseminated intravascular coagulation^27^.

Ranucci and Panagida performed comprehensive coagulation analyses in critical ill patients with COVID-19 including viscoelastic testing and demonstrated increased clot firmness beside significant elevations in levels of d-dimers and fibrinogen-levels as reported by various other authors^28–30^. The amplitude of the d-dimer level was associated with increased mortality in several studies^30,31^. Spiezia et al. and Pavoni et al. have also recently shown severe hypercoagulopathy in critical-ill COVID-19 patients using viscoelastic testing^32,33^. Microthrombus formation in the lungs and various other organs has been described in autopsy series^11,34^ Microvascular injury associated with complement deposition might serve as a possible explanation as Magro et al. reported in their study^35^.

SARS-CoV2 can infect endothelial cells through the ACE2-receptor and cause endothelial damage and apoptosis^36^. Endothelial injury resulting in substantial endothelitis together with dysfunction seems to play a crucial role in the induction of microvascular thrombosis in COVID-19^37^.

Panagida et al. found diminished activity of fibrinolysis in their ROTEM-analysis^28^. Similar changes have been reported in septic patients and might indicate protective mechanisms employed to isolate intruding pathogens^38,39^. One might interpret the persisting fibrinolytic shutdown in COVID-19 as a consequence of the fact that there is no effective therapeutic agent to influence viremia until today and to protect the endothelial cells that are not only the target of SARS-Cov2 but also the key tissue regulating fibrinolysis. Impaired fibrinolysis has also been linked to the pathogenesis of ARDS in general^40,41^.

Continuosly reported high levels of inflammatory cytokines and infiltration of tissues with granuloctes and monocytes as demonstrated for lung tissue in autopsy using caspase-3 immunostaining probably also play an important role in the pathogenesis of coagulopathy and thrombophilia in COVID-19^37,42^ with a particular impact on endothelial damage.

Tang et al. demonstrated decreased mortality in COVID-19 patients with coagulopathy who were treated with either unfractionated heparin or low molecular weight heparin (LMWH) for 7 days or longer compared to those who did not receive heparin^43^. Heparin has anti-inflammatory effects and might mitigate capillary leakage and favorably influence the toxic effect of damage associated molecular patterns (DAMPS) and histones on the endothelium^44,45^.

The American College of chest physicians (ACCP) and the American Society of hematology (ASH) recommended low molecular weight heparin (LMWH) in prophylactic doses in critical ill COVID-19 patients and in therapeutic dose if venous thromboembolism (VTE) occurred^19^.

Recent guidelines of the International society on thrombosis and hemostasis (ISTH) recommend either prophylactic or intermediate dosing of LMWH or UFH^46^.

Given the high incidence of thromboembolic events many centers switched to intermediate dosing of anticoagulation instead of standard prophylaxis^26,47^.

As a consequence, our patients received unfractionated heparin with a target PTT of 50–55 (normal range aPTT 39 s) seconds as long as there were no thromboembolic complications. Patients with thromboembolic events received therapeutic doses of unfractionated heparin with a target PTT of 60–80 s. We decided against routine measurements of AT-III-levels in all patients, instead patients who received more than 1800 U of unfractionated heparin per hour and still did not reach their taget PTT were classified as heparin resistant and switched to Argatroban. The reason for this being the fear of increasing the risk of enhancing bleeding episodes by substitution of AT-III while at the same time further uptitrating the dose of unfractionated heparin, given the difficult situation of frequent thromboembolic complications on the one hand and reported enhanced risk of bleeding episodes on the other. We decided to use Argatroban and not Bivalirudin due to the greater experience in our department with the use of Argatroban. We did not encounter spontaneous prolonging of the aPTT and did not routinely check for lupus anticoagulans. Since the turnaround time for the anti-Xa-activity is about 12 h at our institution, we decided against its use for guiding our anticoagulation management.

In contrast to individual parameters, viscoelastic methods like thrombelastography and ROTEM permit functional evaluations of whole blood aggregometry. Thus it allows evaluation of the different and complex coagulation phases including the initiation, formation and stabilization of a clot, and finally, clot lysis. Still endothelial function and the influence of soluble tissue factor have to be taken into account as they will not be represented in the results of the test. Not only bleeding diathesis as the classical indication for viscoelastic testing but also hypercoagulable conditions due to different diseases were examined in the past using ROTEM and states of hyper- and hypofibrinolysis could be reliably detected and characterized by viscoelastic tests^38,48–50^.

Our cohort presented with a significantly increased clot firmness on the one hand and severely impaired fibrinolytic activity represented by a maximum lysis of < 3% during their ICU-stay on the other.

The clot lysis parameter ML provides information on the fibrinolytic capacity and was successfully used in several studies to asses hyper-, or hypofibrinolysis. Lower values of ML provide evidence of existing hypofibrinolysis, while values above 15% are suggestive for hyperfibrinolysis. Other groups already demonstrated that global haemostatic tests in critical ill patients with COVID-19, tests revealed a hypercoagulable state. That holds true for viscoelastic tests like TEG and ROTEM as well as Thrombin Generation (14, 15, 16) and Clot Generation Waveform analysis (17, 18). Nouigier et al. reported in their recent study that critical ill patient with COVID pneumonia have an impaired fibrinolytic capacity which was associated with increased levels of PAI-1 and TAFI^14^. It has also been proposed that decreased activity of urokinase-type plasminogen activator and increased release of plasminogen activator inhibitor-1 might be the mediating mechanism of hypofibrinolysis, but data to support this further are scarce^51^.

On their 12-week follow up visit, clot firmness and fibrinolytic activity had normalized in all patients. The significant increase of ML in the follow-up assessment indicates an appropriate regeneration or reversibility of physiologic fibrinolytic capacity.

To the best of our knowledge, this is a first study reporting follow-up data on the reversibility of thrombelastometry abnormalities after COVID-19. We found normalized fibrinolytic activity and normalized clot firmness. In contrast, von Meijenfeldt et al. found sustained prothrombotic changes in COVID-19 patients 4 months after hospital discharge. 53Hemostasis exams showed enhanced thrombin-generating capacity and decreased plasma fibrinolytic potential. Further studies will be necessary to clarify this contradictory data situation.

ACCP recommends to evaluate patients for extended prophylaxis after their hospital stay depending on their risk of bleeding^19^. ISTH states, that post discharge prophylaxis for 2–6 weeks should be considered^46^. For patients after VTE, current guidelines recommend therapeutic anticoagulation for at least 3 months^46^.

Especially patients with high d-dimer values were found to be at high risk of post-discharge VTE independent of COVID-19^52^. Around 60% of all VTE in medical patients occur in the post‐hospital discharge period with a more than 5 times increased risk in fatal pulmonary embolism^53^. Newer studies reported favorable risk–benefit ratios for extendend prophylaxis in medical patients^54^.

In conclusion our patients who suffered from thromboembolic events during their course of COVID-19 received therapeutic anticoagulation during their ICU stay and continuation was recommended for the first 3 months thereafter. Taking into account the grade of immobilization and the high levels of d-dimers, increased clot firmness and severely impaired fibrinolysis on viscoelastic testing, we recommended to evaluate therapeutic anticoagulation in the patients without thromboembolic complications at least until reassesement during their first visit on the post-ICU-clinic. On the other hand, proper patient selection to identify patients at higher risk for bleeding while at the same time weighing it against the risk of thrombosis is crucial.

On their 3 months follow-up visit all patients presented with normalized values of d-dimer, fibrinogen and viscoelastic testing. Inflammatory markers were also normalized. Since there were no signs of a persistent hypercoagulable state left and none of the patients suffered from a thromboembolic event after discharge the discontinuation of anticoagulation can be discussed and future clinical trials are needed.

Our study has several limitations. We report the data of a relatively small single center cohort of critically ill patients which may limit generalizability. Due to its retrospective nature it can only be hypothesis generating. Our presumptions have to be verified in a clinical trial focusing not only on coagulation profiles but also on clinical data such as rate of thromboembolic events and ideally survival.

Furthermore, in the meantime between ICU discharge and follow-up, no control follow-ups were routinely performed or analyzed.

Moreover, we only performed anamnesis, clinical examination and screening ultrasound examinations as follow-up exams.. There were no clinical or anamnestic hints for pulmonary embolism but routine tests regarding asymptmomatic events were performed so no statements regarding asymptomatic pulmonary embolism can be made.

In summary, we found substantially limited fibrinolysis in acutely ill COVID-19 patients with normalization after three months.

## Conclusion

In our cohort of critically ill COVID-19 patients, the coagulation profile and inflammatory markers were completely normalized three months after discharge from the ICU. It thus appears reasonable that anticoagulation can be discontinued beyond this timepoint in patients with complete venous reperfusion.

## Data Availability

The datasets analyzed during the current study are available from the corresponding author upon reasonable request.

## Abbreviations

ROTEM: Rotational thrombelastometry
COVID-19: Coronavirus 2019
SARS-CoV-2: Severe acute respiratory syndrome coronavirus 2
ARDS: Acute respiratory distress syndrome
ICU: Intensive care units
MCF: Maximum clot firmness
ML: Maximum lysis
PT: Prothrombin time
INR: International normalized ratio
aPTT: Activated partial thromboplastin time
t-PA: Tissue-type plasminogen activator
PAI-1: Plasminogen activator inhibitor-1
IQR: Interquartile range
ROC: Receiver operating characteristic
AUC: Area under the curve
CI: Confidence intervals
PCT: Procalcitonin
CRP: C-reactive protein
